# Aberrations Causing Neurovascular Damage in the Anterior Maxilla during Dental Implant Placement

**DOI:** 10.1155/2017/5969643

**Published:** 2017-07-13

**Authors:** Shane J. J. McCrea

**Affiliations:** The Dental Implant and Gingival-Plastic Surgery Centre, Bournemouth, Dorset BH7 6AF, UK

## Abstract

When dental implants are being considered for placement in the maxillary central incisor region, proximity to the nasopalatine canal and its contents needs to be accounted for. The morphology of the canal changes with age. The availability of CBCT has allowed the in-depth analysis of this important variable anatomy. However, an associated important anatomical structure can be easily overlooked: the “canalis sinuosus.” This is a neurovascular canal carrying the anterior superior alveolar (ASA) nerve and artery. CBCT frequently shows the canalis sinuosus (CS) as a wide canal lateral to the nasal cavity and also under the anterior part of the nasal floor in close proximity to the NPC. The CS distributes both neural supply and vascular supply to the maxillary anterior teeth which on CBCT sagittal analysis are seen as very fine circular canals having nondistinct walls. The author presents a case history of dental implant placement in the anterior maxilla which resulted in neurovascular disturbance as a result of invasion of the nasopalatine duct and injury to its contents together with the unidentified injury to an aberrant well-defined neurovascular canal inferior to the canalis sinuosus.

## 1. Introduction

Successful dental implants in the anterior maxilla are determined by the available bone and neighbouring neurovascular structures: the nasopalatine canal, the incisal foramen, and the* canalis sinuosus *[1–3].

Following tooth extraction, the maxillary alveolus suffers buccolabial wall resorption and loss of alveolar height: [4] this is progressive throughout life. The nasopalatine duct (NPD) may become so superficial that its contents emerge from the alveolar ridge crest [5]. Additionally, the nasopalatine canal tends to enlarge in all dimensions after tooth extraction and progressively with age [6]. Further, migration of the NPD in the direction of an edentulous ridge is not unusual. Availability of CBCT allows in-depth analysis of these variants.

**Figure 1:**
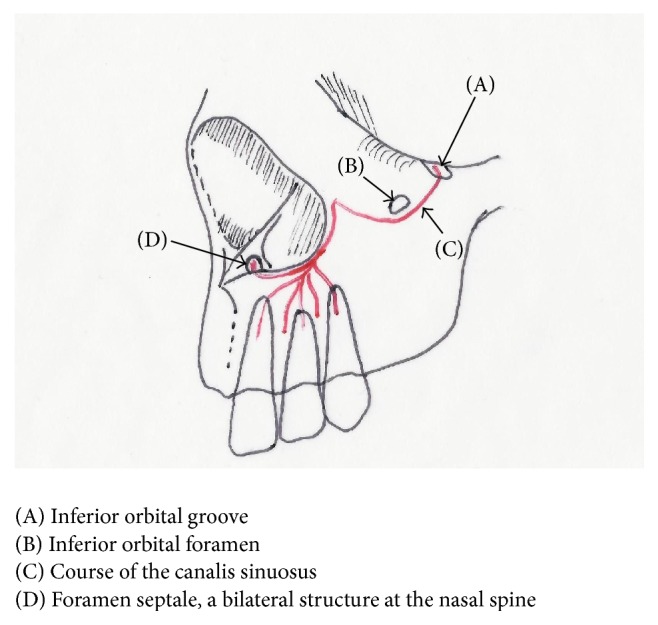
Schematic of maxilla describing the course of the* canalis sinuosus*.

The* “canalis sinuosus”* [7] carries the anterior superior alveolar (ASA) nerve and artery [8]. The canal passes downward and medially below the infraorbital foramen [9, 10] and then along the narial margin giving off neurovascular branches, forming a dental plexus in the alveolus supplying the anterior teeth. The terminal branch of the ASA nerve and artery supply the nasal septum via the* foramen septale* (Figure 1). CBCT can show the* canalis sinuosus* as a wide canal lateral to the nasal cavity and also under the anterior part of the nasal floor [11].

Neurosensory disturbance and haemorrhage have been reported during dental implant placement [12–14]. Foramina and canals are a frequent occurrence in the anterior region of maxilla [9, 15]. CBCT technology can offer the implant surgeon not only the chance of increased accuracy but also the avoidance of surgical and restorative complications [16] However, implant surgeons need to be aware of these variations to identify them during CBCT planning. A case history of dental implant placement in the anterior maxilla with invasion of the nasopalatine duct together with the unidentified injury to an aberrant neurovascular canal inferior to the* canalis sinuosus* is presented.

## 2. Case Presentation

A 55-year-old Caucasian female patient had a dental implant placed into site 21 (FDI-Notation) 22 months previously. Prior to surgery, the central incisor and lateral incisor had been missing for more than 20 years, both being replaced with a single-pontic cantilever bridge from the then present upper right canine tooth (23). Tooth 23 had suffered root canal therapy and an apicectomy being the subject of multiple periods of chronic/acute infection. A preoperative CBCT investigation had been carried out. Subsequently, 23 was removed, bone augmentation was carried out, and an implant was placed in position 21. The patient reported profuse postoperative nasal bleeding. During the 6-month postoperative period, she had frequent episodes of nasal bleeding, pain, subnasal swelling, a sense of “blockage,” and “ethmoidal sinusitis.” She was prescribed multiple doses of antibiotics during these periods. With the persistence of her symptoms, the implant surgeon carried out further postsurgical radiographic investigations that included periapical radiographs and a CBCT examination at 5 months postoperatively: no causative diagnosis was determined. The implant was restored regardless of the continuing postoperative symptoms. The patient was subsequently referred to both ENT and Oral and Maxillofacial departments of her local hospitals—neither speciality could determine the cause of the symptoms.

**Figure 2:**
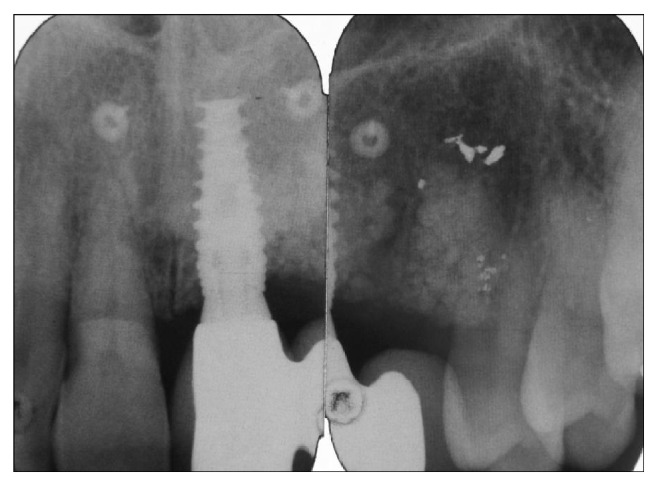
Periapical radiographs taken at initial consultation, showing a restored dental implant in position 21 (FDI-Notation) with no obvious signs of peri-implantitis. That restored implant carried a two-crown distal cantilever bridge.

**Figure 3:**
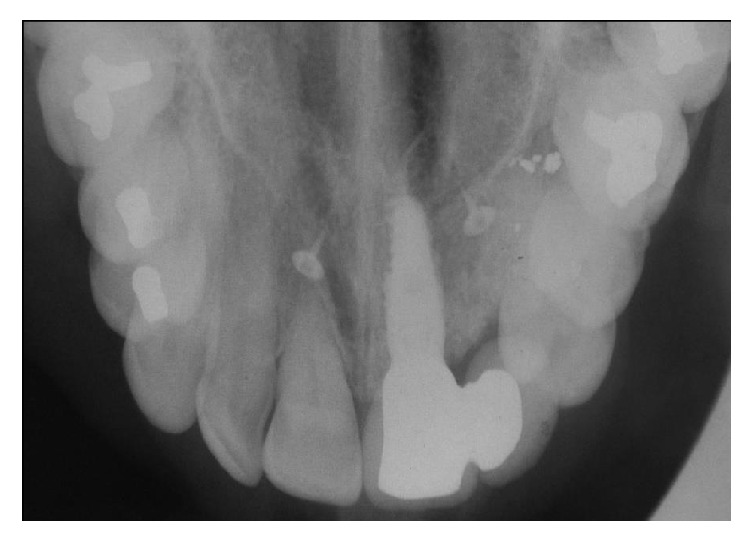
Standard occlusal radiograph taken at initial consultation. The orifices of bilateral nasopalatine canals can be seen clearly apical to the central incisor teeth—the incisive canals: one at the apex of the implant and the other at the apex of natural tooth 11.

**Figure 4:**
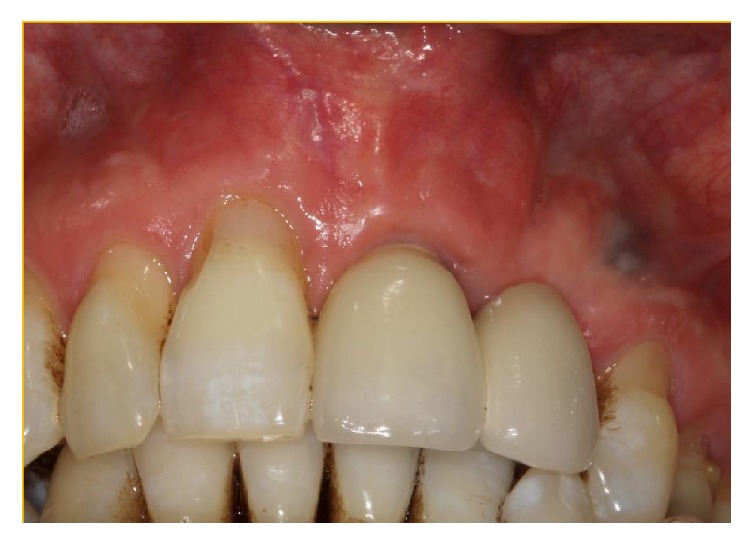
Clinical presentation at initial consultation displaying the erythematous buccal mucosa at implant position 21 (FDI-Notation).

The initial clinical and radiological examination by this author included a standard periapical and an occlusal film (see Figures 2, 3, and 4). The original implant clinic was asked to supply the preoperative and postoperative CBCT scans for further analysis.

### 2.1. Analysis of Preoperative CBCT

**Figure 5:**
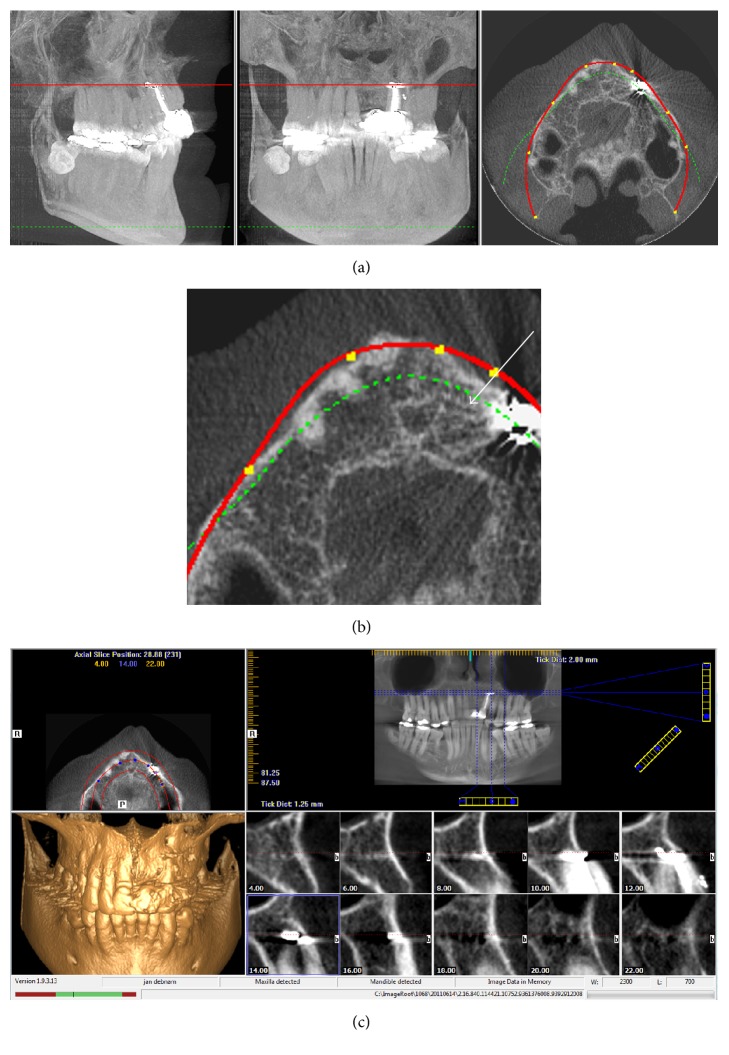
(a) CBCT (iCAT) imagery taken during original implant assessment by implant clinic. (b) Magnified axial image from (a) showing that a well-demarcated canal is present between the apex of tooth 23 and the nasopalatine canal, white arrow. (c) Multiple foramina are easily seen throughout the apical/nasal region prior to implant placement.

To establish the adjacent anatomy and any preexisting pathology, Figure 5(a) shows a lower axial slice at the level of the apex of the apicected tooth 22. Here, a distinct anomaly is seen and a fully differentiated canal is present between the nasopalatine canal/duct and the apicected region of tooth 23 (Figure 5(b)).  Figure 5(c) shows the very distinctive multiple foramina in the nasal region.

### 2.2. Analysis of the Postoperative CBCT

**Figure 6:**
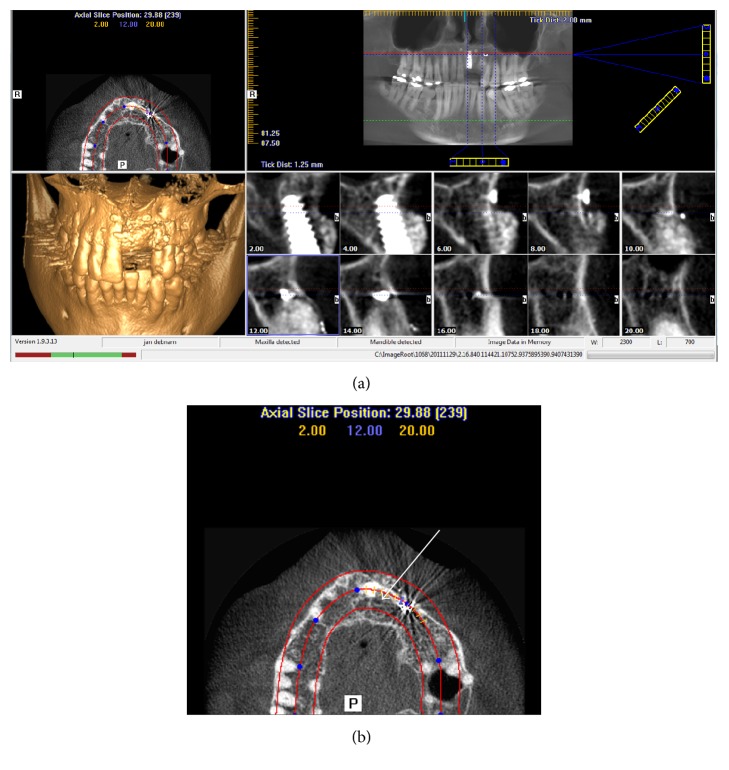
(a) Postoperative CBCT (iCAT) imagery taken at implant clinic to investigate for pathology at osteotomy site 21. The cross-sectional views show that the implant has penetrated the anterolateral wall of the nasopalatine duct. Additionally, the xenograft granules are shown as remaining separate from the alveolus buccal to the implant. (b) Magnified axial image from (a) again showing that a well-demarcated canal is present between the apex of tooth 23 and the nasopalatine duct and palatally to the newly placed implant, white arrow.

Figure 6(a) shows radiated views through the area of interest, showing that the implant is placed through the anterolateral wall of the nasopalatine canal/duct structure and the opening to the lateral aberration (Figure 6(b)). Multiple foramina are present through the nasal floor.

### 2.3. Management

**Figure 7:**
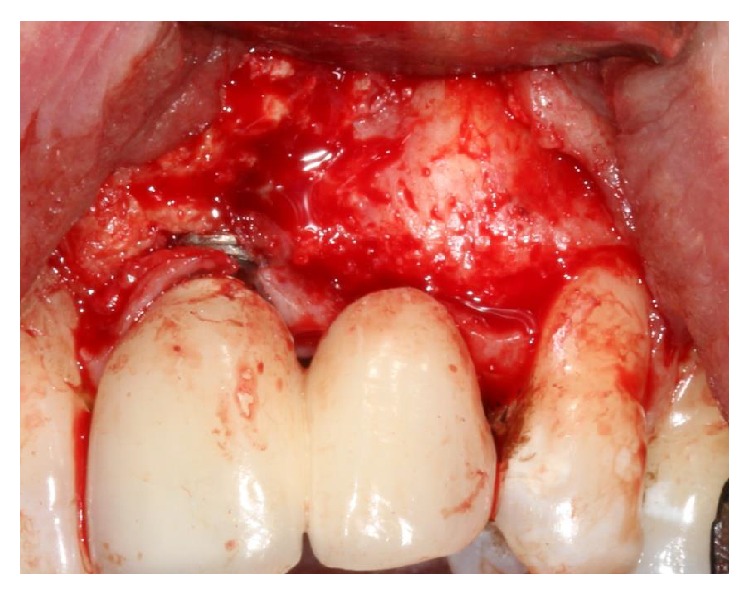
Classical labial “glove” reflection of buccal mucosa displaying the lack of osteogenesis of the xenograft at the buccodistal surface of the implant in position 21 with the lack of coverage of the implant surface.

**Figure 8:**
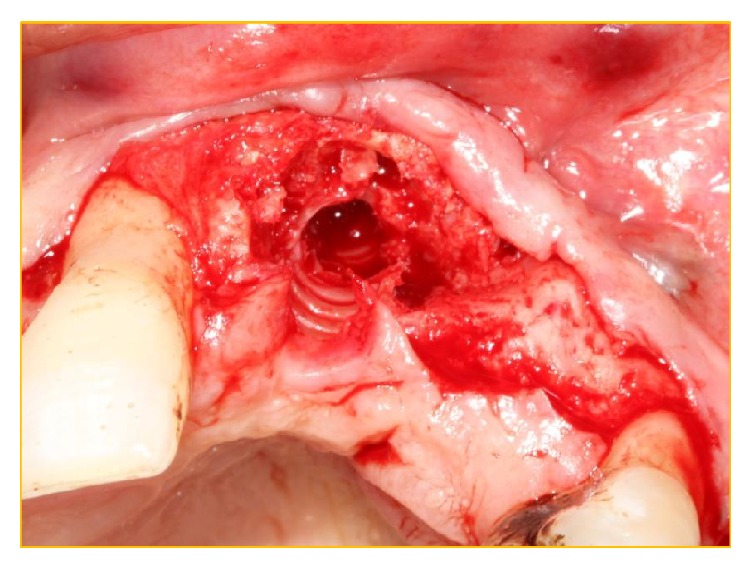
The implant has been simply “unscrewed” with minimal removal torque applied via Adams Orthodontic Pliers.

**Figure 9:**
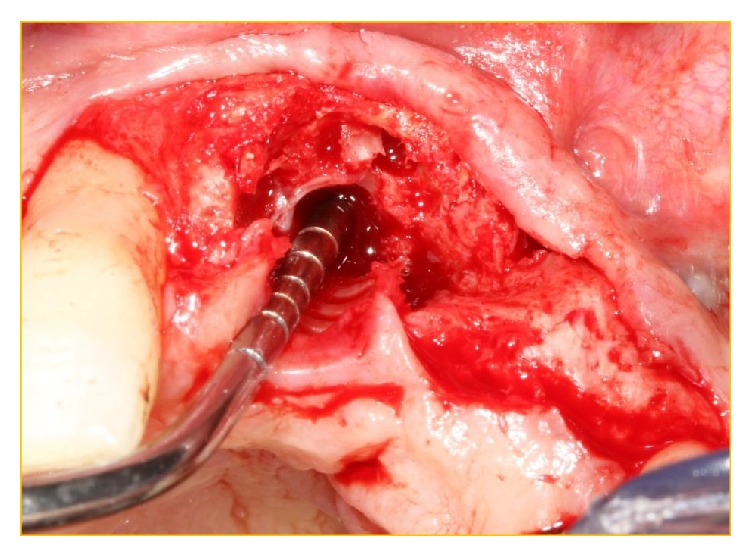
Probing of the implantotomy site to demonstrate that the apical portion was in the nonresistant nasopalatine canal.

**Figure 10:**
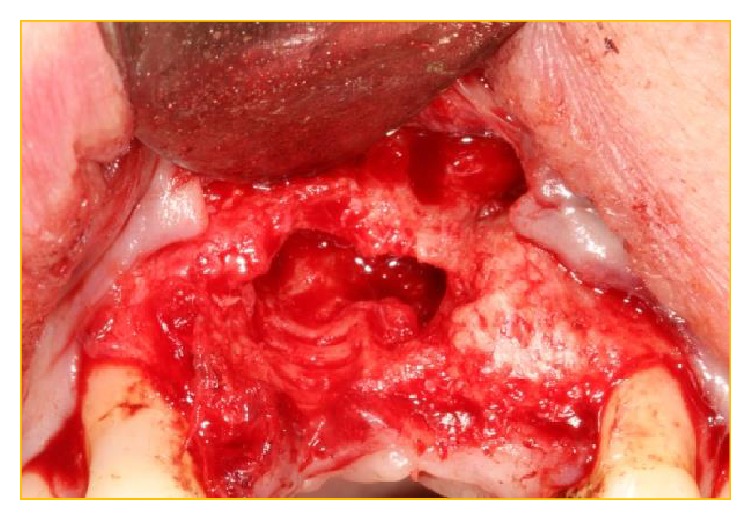
The xenograft was easily debrided from the osteotomy site to display the very patent aberrant canal present between the nasopalatine duct and the aberrant vascular canal.

**Figure 11:**
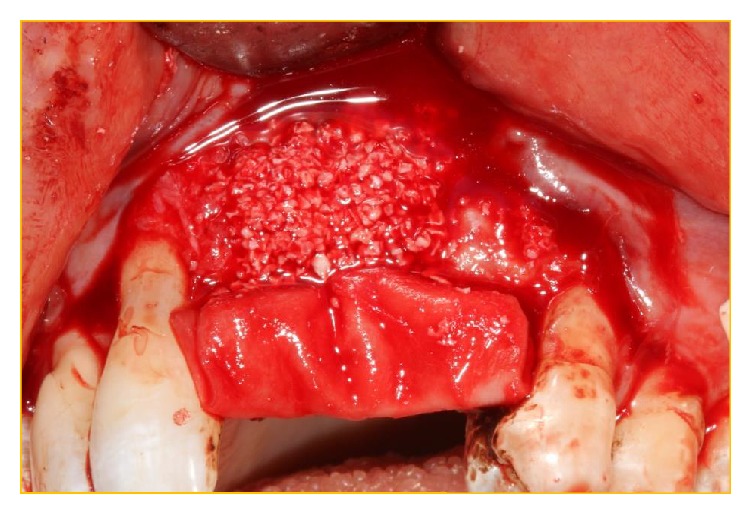
The membrane Bio-Gide has been secured under the palatal tissue at the operative site. Bio-Gide has also been placed at the perforation of the NPD and the distal opening of the conduit inferior to the multiple foramina. The xenograft Bio-Oss has been packed into the “conduit” and then used to augment the deficient buccal bony surface.

**Figure 12:**
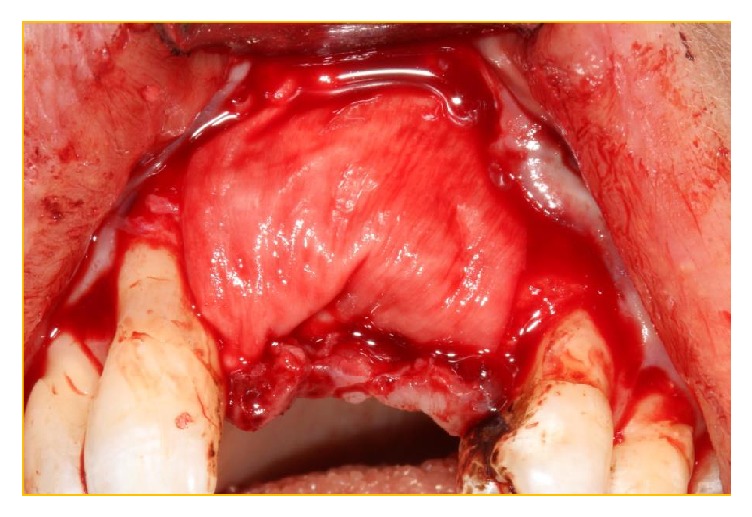
Bio-Gide has been folded over the Bio-Oss granules.

**Figure 13:**
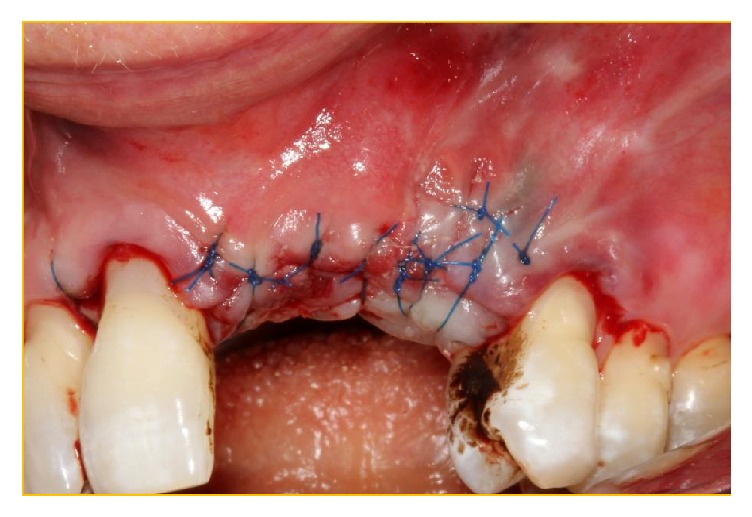
The “labial glove” has been replaced and secured with interrupted 5.0 PROLENE sutures.

**Figure 14:**
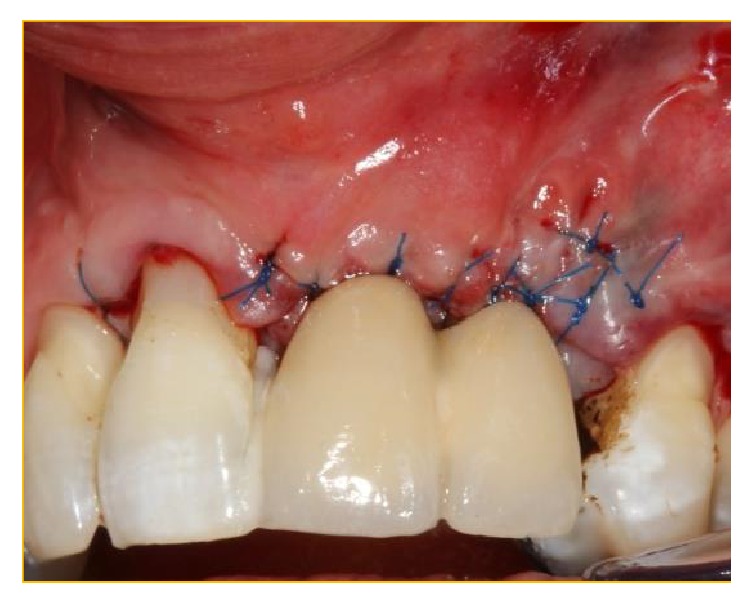
Temporary adhesive bridge has been placed, replacing teeth 21 and 22 and protecting the surgical site.

Implantotomy was carried out under antibiotic cover and intravenous sedation. Following implantotomy and complete debridement (Figures 7, 8, 9, and 10), Bio-Gide was placed apically and posteriorly in the conduit at the site of the multiple descending branches of* canalis sinuosus *to form a barrier. Bio-Gide was also placed over the contents of the nasopalatine canal. Xenograft granules (0.25–0.5 mm Bio-Oss, Geistlich Pharma, Wolhusen, Switzerland) were used to fully obturate the conduit and the surgical defect (Figures 11, 12, and 13). A temporary adhesive bridge was fitted (Figure 14). During follow-up, it was noted that nasal bleeding and sinusitis were arrested and the sensation of nasal “blockage” disappeared “immediately.” The remedial surgical site remains symptomless.

**Figure 15:**
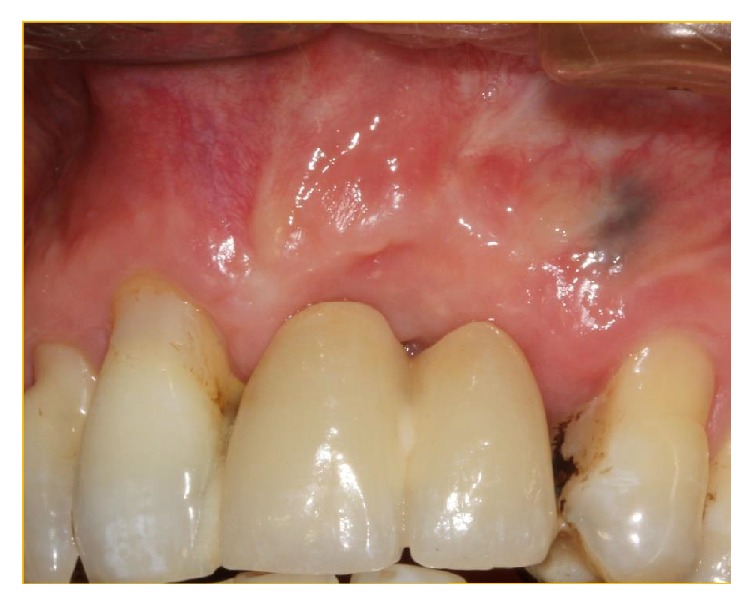
Surgical site 8 months postoperatively.

**Figure 16:**
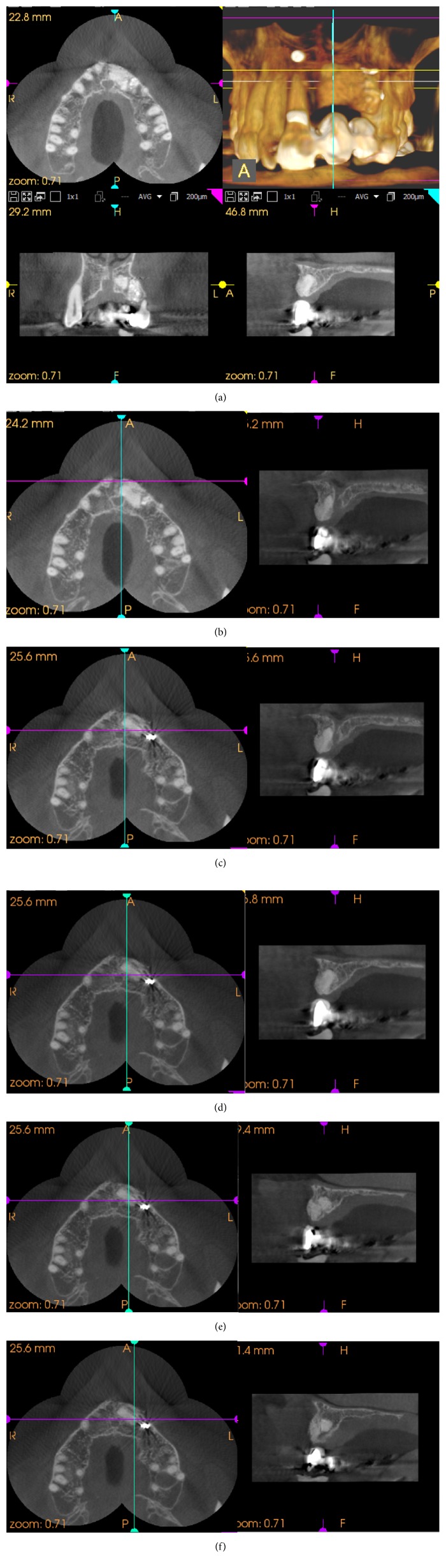
(a) CBCT (Carestream CS9000) imagery taken at 8 months postoperatively displaying the extent of the newly integrating xenograft. (b)–(f) The sagittal views of these films show the well-defined morphology of the aberrant neurovascular canal.

Postimplantotomy CBCT (CS9000, Carestream, Paris, France.) imagery taken at 8 months after surgery determined the degree of osteogenesis and the gross morphology of the operative site (Figures 15 and 16(a)). Figures 16(b)–16(f) show sagittal images that are a progressive medial to lateral display of the well-rounded distal aspect of the former neurovascular canal.

## 3. Discussion

Migration of the NPD in the direction of an edentulous ridge will occur with time. Further, Thakur et al. in a recent study analysing the anatomy and morphology of the nasopalatine canal showed that the size of the NPD increased in the edentulous ridge [17].

The original CBCT scan demonstrated clearly that prior to the original surgery the NPD was no longer in the midline; additionally, two further anatomical variations existed: multiple foramina through the nasal floor superior to and connected to a well-demarcated canal/conduit connecting the former apicectomy site with the nasopalatine duct.

Mraiwa et al. [2] and Liang et al. [18] have reported the existence of up to four foramina through the nasal floor, with Sicher [19] finding up to six separate foramina: these additional foramina have been termed the foramina of Scarpa.

For the case in question, postoperatively, the CBCT scans show the penetration of the nasopalatine duct and the very close proximity of the multiple nasal floor foramina. Excessive bleeding from surgical procedures in the anterior maxilla with unidentified cause and diffuse pain have already been recorded [14]. Balaji feels that damage to the neurovascular content of the* canalis sinuosus* or related anatomical variations must be considered when bleeding and pain exist [9]. Here, removal of the implant and the repair of the anatomy were advocated with obliteration of the arterial conduit between the vascular plexus and the nasopalatine duct using particulate xenograft.

Preoperative identification of anatomical variations, especially those involving neurovascular structures, plays an all-important role in producing successful outcomes for surgical procedures in the anterior maxilla. The AAOMR recommends that cross-sectional imaging be used for the assessment of all dental implant sites and that CBCT is the imaging modality of choice for gaining information. However, such radiological examinations must be individually justified [20]. CBCT should be considered, where a 3D anatomical representation will appreciably enhance the information from implant osteotomy or bone augmentation sites [21]. This presented case reinforces the need for the understanding of the anterior maxillary anatomy [15]. Anatomical variations in the neurovascular plexus need to be recognised, since they may well have an influence on surgical delivery to that site, which in this case would be that of successful implant placement. If postoperative bleeding and paraesthesia ensue over a long period following dental implant placement in the anterior maxilla, it must be postulated that neurovascular damage has occurred. The identification of the damage to the canalis sinuosus, its vascular plexus, or any aberrant canals will facilitate treatment.
